# Study on the benefit analysis based on whole life cycle carbon emission calculation after the construction of photovoltaic systems in macau's construction waste landfills

**DOI:** 10.1038/s41598-024-56803-x

**Published:** 2024-03-30

**Authors:** Zhaobin Li, Waifan Tang, Shulun Mak, Qingwen Li, Jiena Yu, Haolin Chen

**Affiliations:** Department of Construction and Quality Management, School of Science and Technology, HongKong Metropolitan University, Homantin Kowloon, 999077 HongKong SAR China

**Keywords:** Macau construction waste landfill, Photovoltaic system, Life cycle analysis, Environmental economics, Environmental sciences, Engineering

## Abstract

This study seeks to assess both environmental and economic effects associated with installing photovoltaic systems within construction waste landfills in Macau by employing an effective carbon emissions calculation methodology and benefit analysis method. Beginning by outlining characteristics and challenges associated with construction waste landfills, as well as photovoltaic systems used for this application in this paper. Here, we present a detailed outline of our methodology design, outlining its principles of life cycle analysis, data collection processes and the creation of carbon emissions calculation models. Subsequently, we examine photovoltaic systems within Macau's construction waste landfills by studying system design, component selection and operational strategies as well as carbon emission data collection during their operational time period. Under life cycle carbon emissions calculations, we assess the carbon emissions generated from photovoltaic systems as well as conduct an environmental and economic benefit analysis for carbon reduction benefit analysis purposes. This research incorporates sensitivity analysis and uncertainty consideration in order to conduct an extensive benefit analysis. The research results offer strong support for sustainable photovoltaic systems within Macau waste landfills as well as insights to inform planning and policy formation for similar future projects.

## Introduction

### Background and research motivation

In response to escalating concerns regarding global climate change, the imperative for renewable energy has become a pivotal factor in mitigating carbon emissions and promoting sustainable development. Solar photovoltaic systems, recognized as environmentally friendly energy technology, have garnered significant attention and widespread implementation.

Nevertheless, the construction and operation of these systems entail the potential for substantial environmental and resource implications, particularly in unique settings such as construction waste landfills. Macau, designated as a special administrative region, is presently undergoing intensive urbanization and construction activities, leading to the significant accumulation of construction waste.

These waste landfills, often viewed as formidable environmental and sustainability challenges, pose potential adverse effects on soil, water quality, and atmospheric conditions. Against this backdrop, the integration of photovoltaic systems into the construction of waste landfills aims not only to reduce dependence on conventional energy sources but also holds the promise of transforming and repurposing these landfill areas. This initiative aligns with the imperative to address environmental challenges while fostering sustainable development.

### Research objectives and problem statement

This research endeavors to scrutinize the environmental benefits associated with integrating photovoltaic systems into Macau's construction waste landfills, utilizing life cycle carbon emissions calculations. The primary objective involves a comprehensive exploration of these advantages, specifically focusing on the following dimensions:

1. Examine life cycle carbon emissions associated with the integration of photovoltaic systems within Macau's construction waste landfills.

2. Investigate sustainable design and management strategies, providing insights to landfill operators to optimize the installation of photovoltaic systems within waste sites.

This research aims to offer crucial insights into installing photovoltaic systems within construction waste landfills in Macau and similar urban special regions. The overarching goal is to align with objectives related to carbon reduction, resource reuse, and sustainable urban development.

## Literature review

### Construction waste landfills

Characteristics and Challenges of Construction Waste Landfills Construction waste landfills are sites accumulating construction and demolition waste such as concrete, bricks, wood or any other building material from demolition work projects. Such landfills pose various characteristics and challenges such as:

1. *Waste diversity* Construction waste landfills typically hold waste materials generated during both demolition projects and construction worksites, including concrete fragments, bricks, wood, metals, glass and plastics—such as those from concrete fragments to bricks to wood and metal to wood composites to non-uniform soil properties in terms of its composition resulting from interactions among various materials that result in nonuniform soil properties and an array of varying composition. Understanding of types, distribution and characteristics of waste are integral in designing photovoltaic systems as compaction levels thermal conductivity moisture absorption characteristics as well as compaction levels among materials may impact upon design of support structures as well as foundation stability issues that need to be considered when designing photovoltaic support structures as well as stability of foundations^1^.

2. *Soil quality in waste landfills* Soil quality at waste landfills may be affected by their waste composition, with various waste types potentially emitting hazardous substances, including heavy metals or organic compounds into the ground that pose threats to sustainability and environmental impacts of photovoltaic systems. Therefore, comprehensive analysis and monitoring must take place regularly in order to protect and stabilize landfill soils—particularly during periods of precipitation drainage which could release potentially toxic compounds into the air which could further impact upon its integrity and negatively affect our planet's sustainability environmental impacts^2^ .

Research should take a holistic approach when investigating waste diversity and soil quality issues, including categorizing waste for treatment and testing as well as soil improvement methods. Such practices will assist designers of photovoltaic systems within waste landfills to optimize system stability, performance and sustainability while protecting against hazardous substance contamination during construction or operation of photovoltaic systems. Likewise, appropriate environmental protection measures must also be put in place in order to limit risks of soil and groundwater contamination by hazardous materials during this process^3–6^.

### Application of photovoltaic systems in waste landfills and sustainability research

Photovoltaic systems installed at waste landfills may offer numerous environmental and sustainability advantages, helping address some key concerns:

1. *Land reutilization* Waste landfills often lie on the outskirts of urban areas and using these areas for photovoltaic systems can make maximum use out of abandoned land. Abandoned construction waste landfills often pose environmental concerns; due to the presence of hazardous materials like trash and waste products, such areas may not be appropriate for traditional land usage. Land reutilization poses the challenge of turning abandoned areas into sustainable and economically beneficial spaces. Photovoltaic system construction offers an effective means of land reclamation without needing extensive restoration efforts, effectively turning abandoned land into renewable energy production locations that bring environmental, energy production and economic benefits simultaneously^7^. By turning abandoned lands into productive spaces for renewable energy production, sustainability in terms of environmental preservation, energy generation and economic benefits can be reached simultaneously.

2. *Carbon emission reductions* Solar photovoltaic systems produce clean electricity that lowers carbon emissions by cutting back on fossil fuel use and dependence, thus decreasing dependence and thus emissions of harmful greenhouse gases such as methane gas^8^. Waste landfills can be considered sources of carbon emissions as they release greenhouse gases such as carbon dioxide and methane during decomposition processes. Installing photovoltaic systems in these areas can drastically decrease emissions of greenhouse gases and help mitigate global warming^9^. Carbon emissions generated during photovoltaic system construction and operation tend to be relatively minimal when compared with fossil-fueled electricity generation, particularly landfill installation of photovoltaics which has proven particularly successful at mitigating climate change effects while simultaneously contributing towards economic and societal wellbeing.

3. *Resource reuse* Producing photovoltaic components typically involves various materials that may come from waste landfills—providing opportunities for resource reuse^10^. Construction waste landfills contain underutilized resources like concrete, steel, glass and plastics that have gone unused—costing the environment as well as valuable resources that would otherwise remain unspent. Photovoltaic system construction offers an opportunity to repurpose waste resources, reduce waste production and increase resource sustainability—while at the same time improving their economic viability and economic feasibility of photovoltaic system projects. By effectively recycling abandoned resources it's possible to lessen environmental impact while decreasing waste generation while simultaneously improving resource use efficiency and improving economic feasibility of photovoltaic system projects^11^.

Photovoltaic systems present many unique challenges when applied in these unique locations, including soil contamination, array design complexity, equipment maintenance costs and ecological effects. Thus, in-depth research must be performed in order to develop sustainable reuse plans for waste landfills.

### Life cycle analysis and carbon emission calculations

Life cycle analysis (LCA) is an environmental impact evaluation method. When applied to solar photovoltaic research, LCA becomes an invaluable resource in terms of measuring carbon emissions, energy efficiency and sustainability—key aspects that evolve alongside research efforts on photovoltaic system technologies^12^. LCA research in these areas:

1. *Enhancing LCA methods* Researchers are constantly making improvements to LCA methods—such as data collection, model complexity and result accuracy—in order to provide more comprehensive assessments of carbon emissions.

2. *Life cycle cost analysis (LCCA)* LCA has evolved over time to incorporate life cycle cost analyses that take account of both environmental and economic benefits^13^.

3. *Carbon neutrality and negative carbon emissions* Some research is being done in an attempt to achieve net zero or negative carbon emissions with carbon neutrality and negative emission strategies.

These cutting-edge research areas will be applied in this study in order to evaluate carbon emissions and sustainability benefits of photovoltaic systems installed within Macau construction waste landfills, providing methodology support for our research project.

## Methodology

### Basic principles and methods of life cycle analysis

The analysis framework for LCA is a systematic approach aimed at comprehensively assessing the environmental impact of a product or system, particularly in the fields of construction and materials. Guided by ISO 14044 standards or other relevant guidelines, the LCA analysis framework encompasses several key steps:

*Goal definition* Initially, it involves precisely defining the objectives of the LCA, including specifying the analysis's purpose, determining the functional unit (i.e., the specific quantity or quality of the product or service under assessment), outlining life cycle stages (spanning from raw material acquisition to disposal), setting boundaries (system boundaries, incorporating considered processes and input–output), and selecting appropriate impact categories. This step is crucial to ensuring the accuracy and comparability of the analysis.

*Life cycle inventory* This step entails establishing a detailed inventory of all processes, activities, and material/energy flows within the system, covering raw material acquisition, production, transportation, use, and disposal. This aids in gaining a comprehensive understanding of the system's composition and energy/material flows, forming the foundation for assessing environmental impacts.

*Data collection* Accurate data collection is vital from various stages within the system, encompassing raw material and energy consumption, emissions, and other relevant parameters. Utilizing reliable data sources is key to ensuring the accuracy of the analysis results.

*Life cycle impact assessment* Building on collected data, this step involves assessing the life cycle impact to calculate various environmental impact indicators. This may include indicators related to climate change, resource utilization, ecological toxicity, providing a comprehensive understanding of the system's overall environmental impact^14^.

*Interpretation and improvement* Following the assessment, results are interpreted, potential improvement opportunities are identified, and sustainable development strategies are formulated. This step goes beyond meeting standard requirements; it aims to translate LCA outcomes into practical environmental improvement measures.

By adhering to ISO 14044 or other relevant standards, the LCA analysis framework offers a systematic and scientific approach, enabling accurate assessment of the environmental performance of products or systems in the fields of construction and materials. The application of this methodology helps guide sustainable design and decision-making, propelling the construction and materials sectors toward more environmentally friendly practices.

### Methods for data collection and analysis

Ensuring the reliability of Life Cycle Assessment (LCA) necessitates the utilization of high-quality and accurate data derived from diverse sources, measurements, and simulations. Various data sources encompass laboratory measurements, literature surveys, and simulation methods. This research will employ the following methods for data collection and analysis^15^:

1. *Field surveys* Conduct on-site surveys to evaluate the present state of Macau's construction waste landfills, considering waste characteristics, soil quality, and environmental conditions.

2. *Literature review* Collect relevant literature to acquire data and information on construction waste, photovoltaic systems, and LCA^16^.

3. *Numerical simulation* Employ specialized software and models for simulating and estimating specific parameters and data, including energy output and carbon emissions.

By employing these comprehensive methods, the research aims to gather robust data, ensuring a thorough analysis of the environmental impact within Macau's construction waste landfill area.

### Establishment of the life cycle carbon emissions calculation model

The establishment of the carbon emissions model for photovoltaic systems based on the Life Cycle Assessment (LCA) method can be represented as follows^14,17–22^:

$${\text{C}}_{{\text{t}}} = {\text{C}}_{{1}} + {\text{C}}_{{2}} + {\text{C}}_{{3}} + {\text{C}}_{{4}} + {\text{C}}_{{5}} + {\text{C}}_{{6}} =_{{{\text{i}} = {1}}}^{{\text{n}}} \;\left( {{\text{E}}_{{\text{i}}} {\text{R}}_{{\text{e}}} } \right) +_{{{\text{i}} = {1}}}^{{\text{n}}} \;{1}\left( {{\text{M}}_{{\text{i}}} {\text{Q}}_{{\text{i}}} {\text{R}}_{{\text{m}}} + {\text{C}}_{{{\text{wc}}}} } \right) \cdot_{{{\text{i}} = {1}}}^{{\text{n}}} \;{\text{W}}_{{{\text{ari}}}} +_{{{\text{i}} = {1}}}^{{\text{n}}} \;\left( {{\text{P}}_{{\text{i}}} {\text{R}}_{{\text{p}}} + {\text{DH}}} \right) \cdot_{{{\text{i}} = {1}}}^{{\text{n}}} \;\left( {{\text{G}}_{{\text{p}}} \eta_{{\text{i}}} {\text{G}}_{{\text{i}}} {\text{R}}_{{\text{g}}} } \right) +_{{{\text{i}} = {1}}}^{{\text{n}}} \;\left( {{\text{M}}_{{\text{i}}} {\text{H}}_{{\text{i}}} {\text{R}}_{{\text{m}}} } \right).$$

C_t_ is the total carbon emissions of the photovoltaic power generation system throughout its life cycle, in kilograms (kg). C_1_ is the carbon emissions during the raw material acquisition phase of the photovoltaic components, in kg. C_2_ is the carbon emissions during the manufacturing phase of the photovoltaic components, in kg. C_3_ is the carbon emissions during the transportation phase of the photovoltaic components, in kg. C_4_ is the carbon emissions during the construction and installation phase, in kg. C_5_ is the carbon emissions during the use and maintenance phase, in kg. C_6_ is the carbon emissions during the decommissioning and cleanup phase, in kg. E_i_ is the electricity consumption for the i-th process, in kilowatt-hours (kWh). R_e_ is the carbon emission factor of electricity, with a value of 0.749 kg CO_2_/(kWh). M_i_ is the energy content of the i-th material per unit, in megajoules per kilogram (MJ/kg). Q_i_ is the quantity of the i-th material used, in kilograms (kg). R_m_ is the conversion factor for energy and carbon emissions for the i-th material, in kg CO_2_/MJ. C_wc_ is the carbon emission factor for aerobic wastewater treatment, in kg/m^3^. Wani is the volume of wastewater treated in each production process, in cubic meters (m^3^). P_i_ is the price of the equipment, in thousands of yuan (¥). R_p_ is the carbon emission factor for specialized equipment in the industrial sector, in kg/ten thousand yuan. D is the transport distance, in kilometers (km). H is the transport mass, in metric tons (t). G_p_ is the global warming potential coefficient of greenhouse gases. η_i_ is the conversion coefficient for converting greenhouse gases into CO_2_. G_i_ is the consumption intensity of fuel oil, in liters per metric ton per kilometer (L/(t·km)). H_i_ is the consumption of the i-th fossil fuel during the construction and installation phase, in kilograms (kg). The energy consumption factor for construction machinery equipment is known. _g_ is the greenhouse gas emission factor for fuel oil, in kg/L, which can be obtained from IPCC (Intergovernmental Panel on Climate Change) guidelines^23^.

Establishing the life cycle carbon emissions calculation model will help us accurately assess the carbon emissions of photovoltaic systems within Macau's construction waste landfills, providing a solid foundation for subsequent analysis^23^.

## Construction of photovoltaic systems in macau's construction waste landfills

### System design and construction process

**Figure 1:**
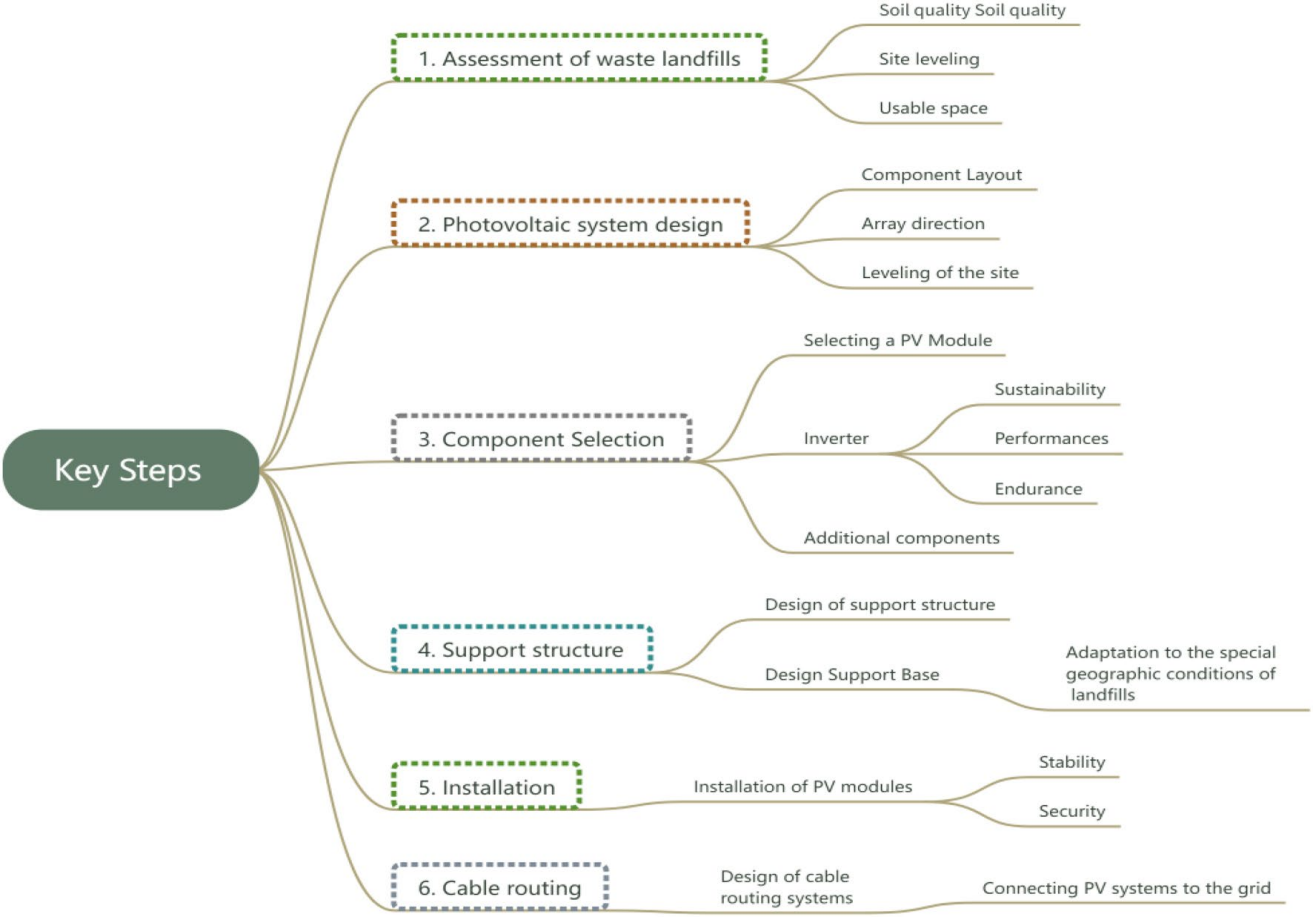
System design and construction process.

The design and construction process of photovoltaic systems within Macau's construction waste landfills requires a comprehensive consideration of waste characteristics, geographical conditions, and sustainability principles^19^. Key steps include (as shown in Fig. 1):

### Component and material selection: sustainability and performance considerations

**Figure 2:**
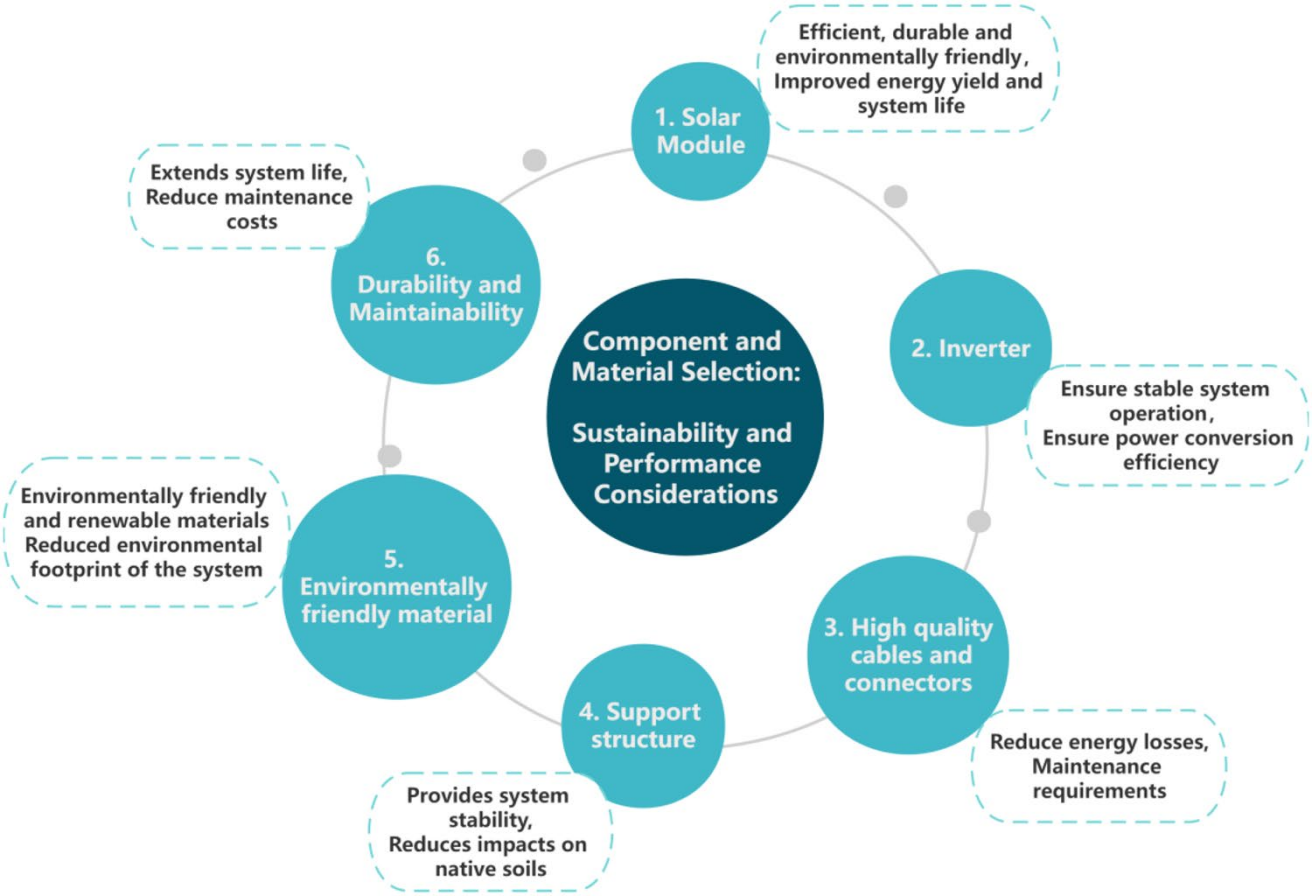
Selection of components and materials.

When selecting components and materials, it is essential to strike a balance between sustainability and performance. Key considerations (as shown in Fig. 2) include^24^:

### Operation and maintenance strategies

**Figure 3:**
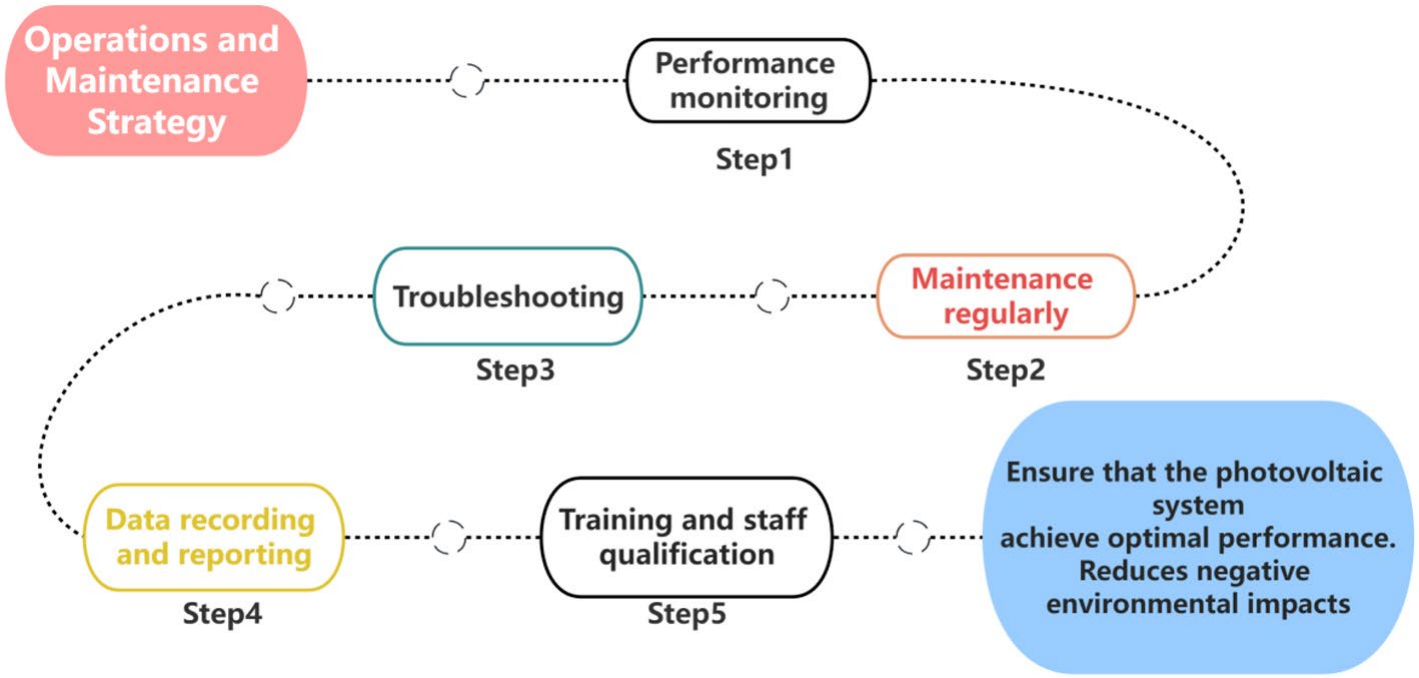
Operations and maintenance policies.

Photovoltaic systems require maintenance and monitoring during their operational phase to ensure sustainability and performance. The following are important components of operation and maintenance strategies (as shown in Fig. 3)^24^:

## Data collection and carbon emission calculation

### Data collection during system operation

**Figure 4:**
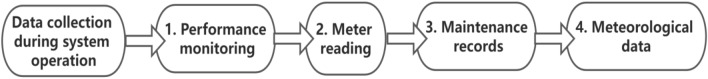
Data collection during system operation.

To perform a full life cycle carbon emission calculation, extensive data collection is required during the system's operational phase. The following are methods for data collection (as shown in Fig. 4):

### Specific methods for carbon emission calculation: components, transportation, energy use, etc

Carbon emission calculation requires a comprehensive consideration of multiple aspects, including the lifecycle of components. This involves calculating the lifecycle carbon emissions of solar modules, inverters, and other components, encompassing production, transportation, and disposal stages^25^.

#### Production stage (as shown in Figs. 5 and 6)

**Figure 5:**
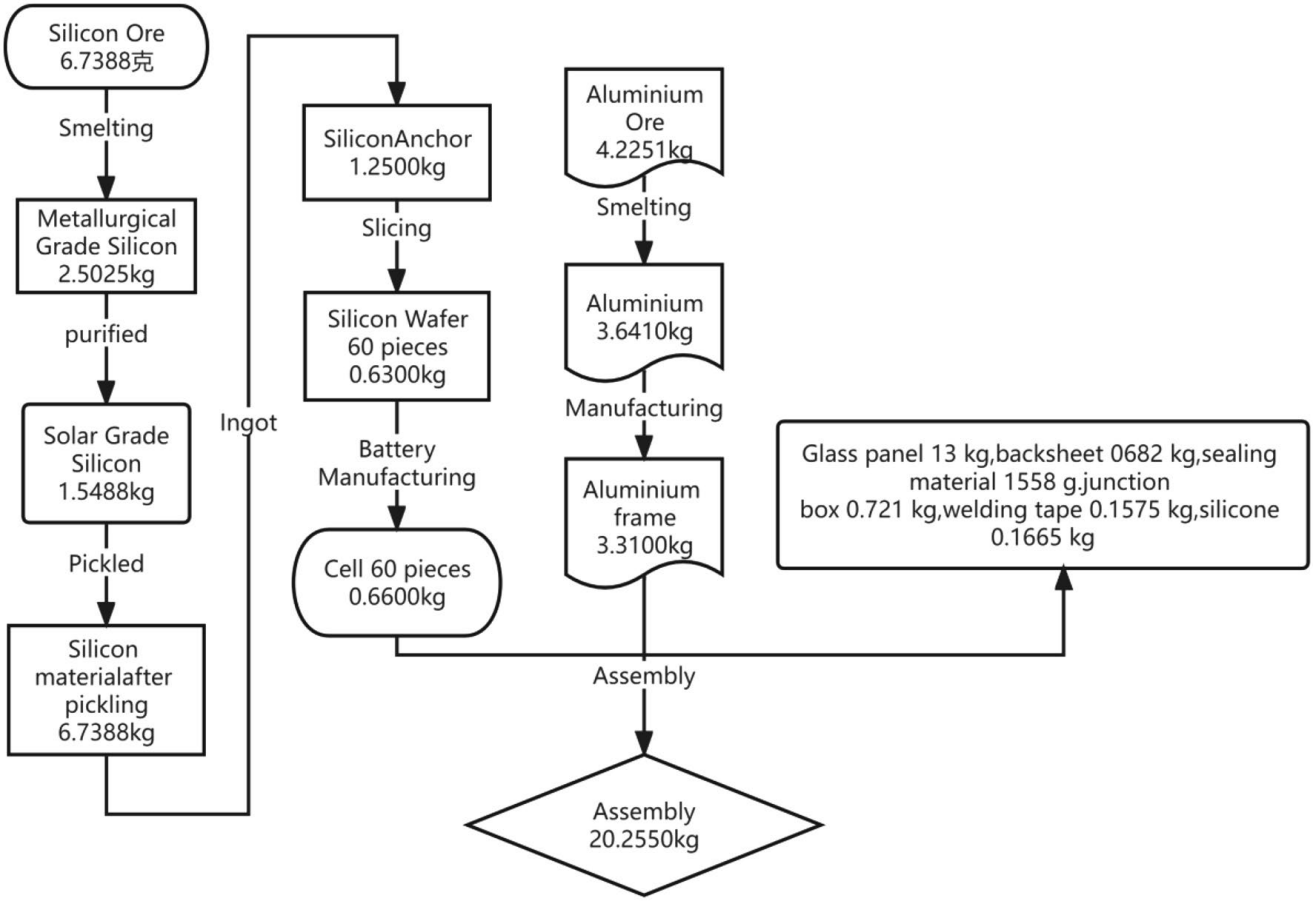
Mass flow of productions.

**Figure 6:**
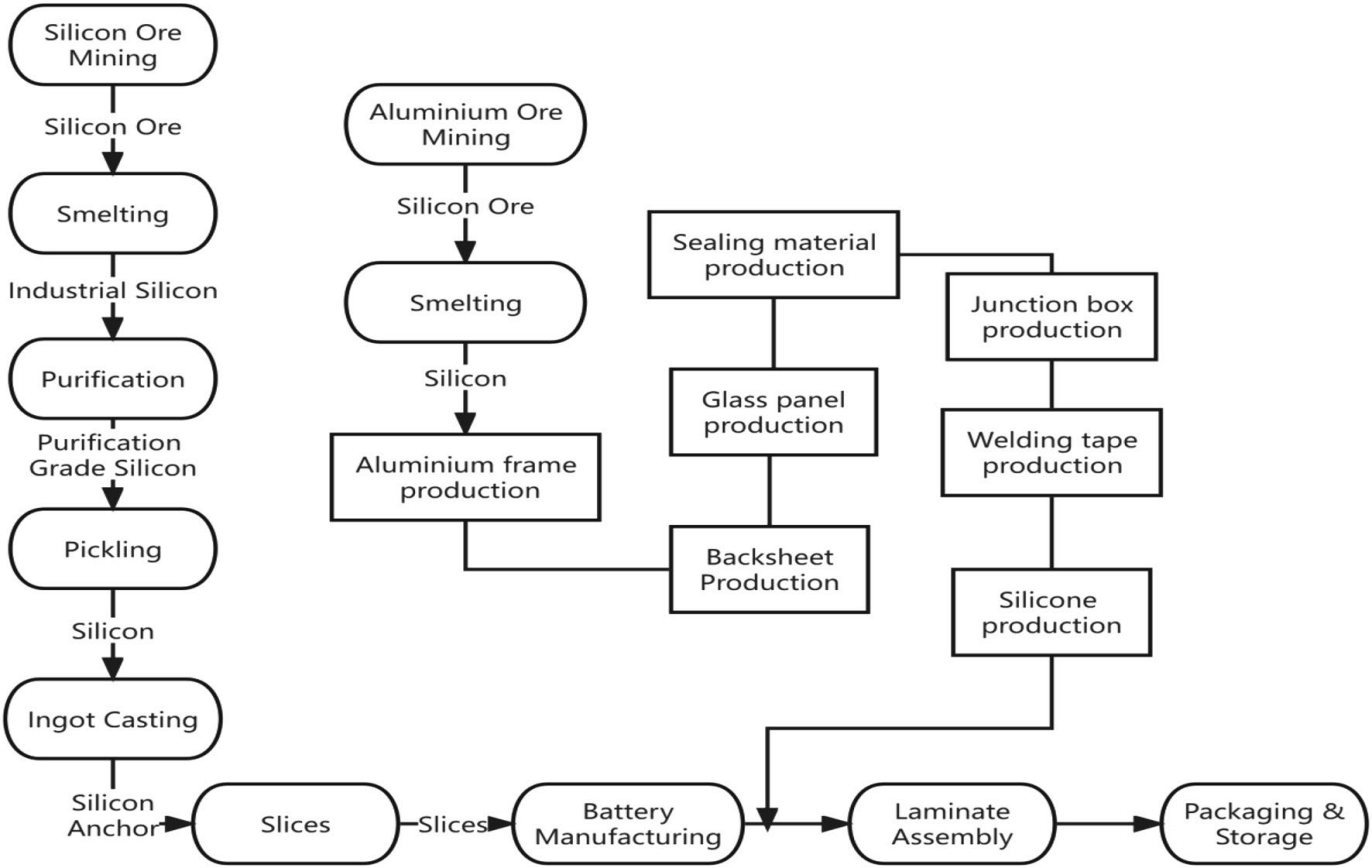
Production schedule of the solar modules.

A. Carbon Emissions in the Production Stage of Photovoltaic Panel Components (Using the Pickling Stage as an Example).

Each stage's energy consumption and data related to transportation, waste disposal, and direct emissions during the manufacturing phase are provided as primary data by the production companies. The calculation data is as shown in Table 1^26^.

**Table 1:** Production phase (a case study of pickling).

Input parameters	Numerical value	Unit	Source
Pickling power consumption	2,050,856.59	kW·h	On-site verification
Total amount of silicon material	3,255,608	kg	On-site verification
Emission factor of electricity required per unit of silicon material for pickling	0.6299	kW·h/kg	Calculated value
Electricity Emission Factor	0.749	kgCO_2_/(kW h)	NDRC document release
Mass of silicon material per module	1.2625	kg	Provided by the manufacturer
CO_2_ emissions per module	0.5957	kgCO_2_	Calculated value

B. Carbon Emissions in the Production Stage of Other Components of the Photovoltaic System (Using Inverters as an Example).

In addition to photovoltaic panel components, the photovoltaic system includes combiner boxes, inverters, etc. The actual usage and specifications are determined based on actual project data. The carbon emission coefficient y of professional equipment changes over time according to the following relationship^27^: y = 2.252e + 143e − 1654x, where x represents the number of years. Carbon emission conversion coefficients are calculated using the 2013 carbon emission coefficient of professional equipment. The calculation data is as shown in Table 2^27^.

**Table 2:** Carbon emissions of other components in the production phase (a case study of inverters).

Input parameters	Numerical value	Unit	Source
100 km fuel consumption of the transport vehicle	20	L/(100 km)	On-site verification
Loading capacity of the means of transport	20	t	On-site verification
Energy consumption per unit of aluminium frame transport	0.01	mL diesel/(kg-km)	Calculated value
Aluminium frames per module	3.31	kg	Provided by manufacturer
Round trip distance for aluminium frame transport	110	kg	Provided by supplier
CO_2_ emission factor for diesel	2.73	kgCO_2_/L	Provided by IPCC
CO_2_ emissions per module	0.0099	kgCO_2_	Calculated value

#### Transportation emissions

Consider the carbon emissions during the transportation of components from the production site to the construction site, including fuel consumption of transport vehicles. The calculation data is as shown in Table 3^28^.

**Table 3:** Transportation phase.

Equipment	Model	Quantity/unit	Unit price/Million Yuan	Carbon emission factor of specialised equipment/(10,000t·billion yuan^−1^)	Carbon emission factor/(kgCO_2_)
Inverter	SG500MX	16	20.75	0.0568	18,857.60
	SG630KTL	1	26.775		1,520.82
	CP500TL	6	26.65		9,082.32

#### Energy use

Estimate the energy requirements during the operational phase of the photovoltaic system to calculate carbon emissions, considering the source of electricity and energy conversion efficiency.

#### Maintenance emissions

Record and assess the carbon emissions from maintenance activities, such as fuel consumption for maintenance vehicles.

Specific carbon emission calculation methods depend on actual data and models. For instance, in the calculation of component lifecycle, it's necessary to consider the carbon emissions from raw material extraction, production processes, transportation, and disposal. In the case of transportation emissions, factors such as transport distance, type of transport vehicle, and fuel efficiency need to be considered. For energy use, factors like the system's electricity generation, grid emission factors, and energy conversion losses need to be considered.

#### Disposal and recycling

The disposal and recycling phase includes carbon emissions from transporting the discarded products and emissions from burying non-recyclable materials. In photovoltaic systems, most component materials are recyclable, and they do not produce emissions in landfills. Therefore, emissions in the disposal and recycling phase for photovoltaic systems are equivalent to transportation emissions. The calculation data is as shown in Table 4^29^.

**Table 4:** Decommission and reclamation phase.

Input parameters	Numerical value	Unit	Source
Diesel consumption per kilometre	0.55	L/km	Provided by NDRC
CO_2_ emission factor for diesel	2.73	kgCO_2_/L	Provided by IPCC
Loading capacity of transport vehicle	10,000	kg	Calculated based on a loading capacity of 10t
Transport emission factor	0.0002	kgCO_2_/(kg.Km)	Calculated value
Calculated value Average transport distance	500	km	Calculated value 500 km
Mass per module	20.255	kg	Supplied by the manufacturer
CO_2_ emissions per module	1.5206	kgCO_2_	Calculated value

### Collection of benefit data

In addition to carbon emission data, benefit data must be collected to evaluate environmental and economic benefits. This includes:

#### Electricity production

**Figure 7:**
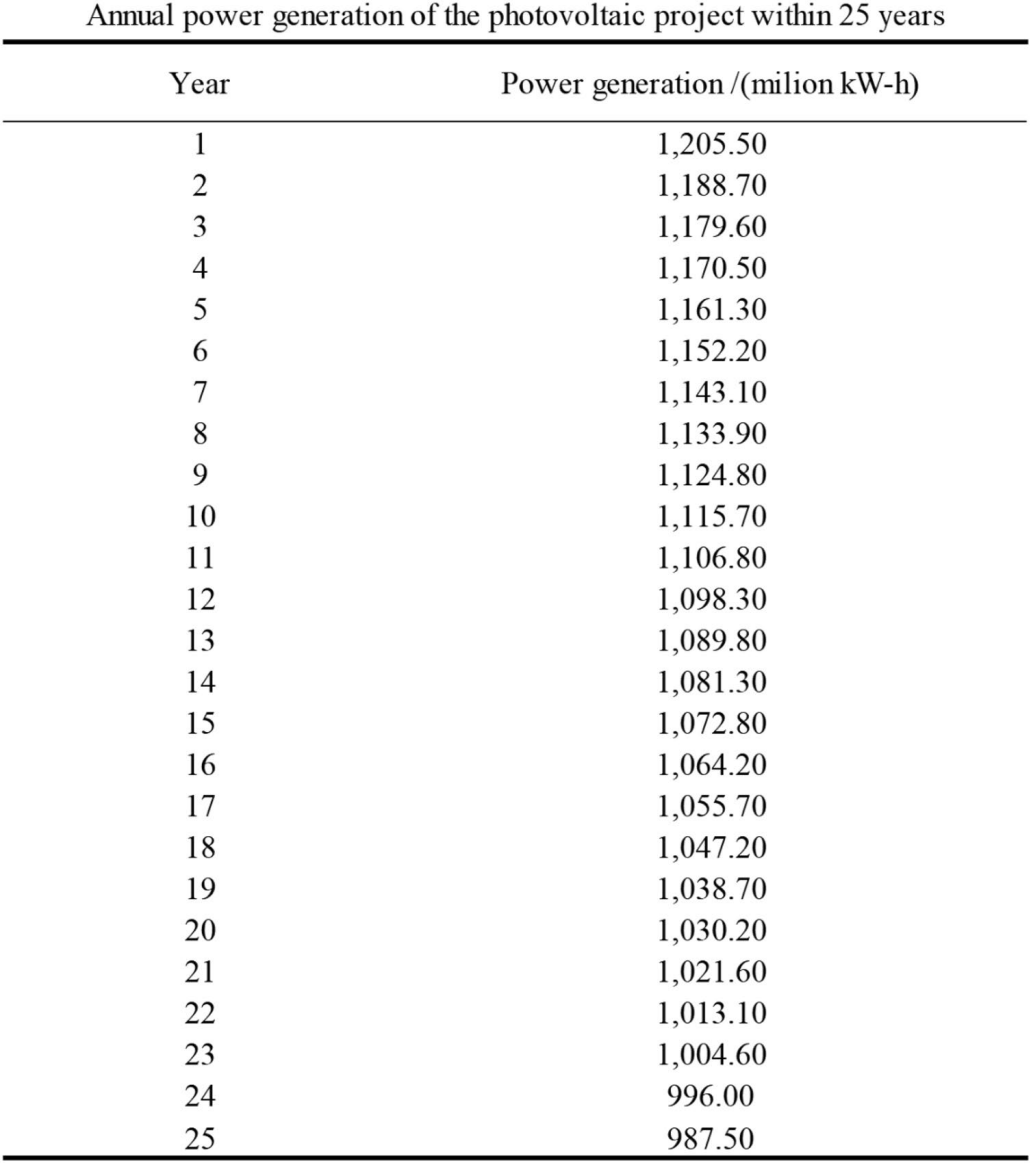
Annual power generation of the photovoltaic project within 25 years.

Record the actual electricity production of the system to determine its performance (as shown in Fig. 7).

From Fig. 7, it can be observed that considering the degradation of the components, the theoretical calculated power generation in the first year is 12.055 million kW/h, which is 10.46% higher than the annual average grid-connected electricity. Following a segmented linear decay, the degradation rate is 2% in the first year and an average annual degradation rate of 0.75% from the 2nd to the 10th year, with a total degradation rate of 6.75%. From the 11th to the 25th year, the average annual degradation rate is 0.7%, resulting in a total degradation rate of 10.5%. The overall lifecycle component degradation rate is 19.25%.

#### Carbon emission reduction benefits

**Figure 8:**
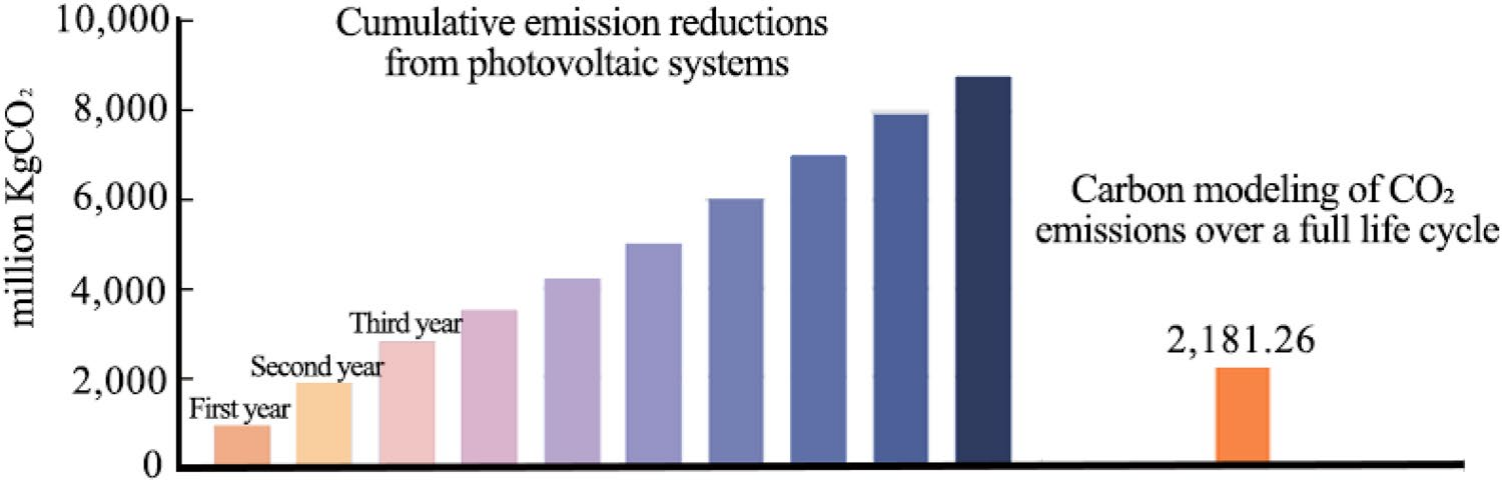
Cumulative emission reductions from photovoltaic systems.

Calculate the carbon emission reductions during the operation of the photovoltaic system, which is the difference in carbon emissions compared to traditional energy sources. Based on the annual grid electricity consumption within the project's lifecycle, the cumulative CO_2_ emission reductions can be calculated annually throughout the project's lifespan. By comparing the total CO_2_ emissions over the entire project's lifecycle, the carbon payback period of the project can be determined (as shown in Fig. 8)^30^.

From Fig. 8, it can be seen that the photovoltaic system's CO_2_ reduction capacity increases linearly with the number of years in operation. The total CO_2_ emissions over the system's entire lifecycle remain constant. In the 2.5th year after the photovoltaic system is put into use, the CO_2_ emission reductions from the system begin to exceed the total CO_2_ emissions over the system's entire lifecycle. Therefore, it can be concluded that the carbon payback period for this project is approximately 2.5 years.

#### Economic benefits

Collect data on investment costs, energy savings, and operational and maintenance costs to assess the economic benefits of the system.

These data will be used for subsequent benefit analysis, helping us understand the sustainability and potential impacts of the photovoltaic system within Macau's construction waste landfill area.

## Discussion

### Calculation of the system's full lifecycle carbon emissions

The calculation aims to provide a thorough understanding of the environmental impact of the photovoltaic system's full lifecycle carbon emissions within the Macau construction waste landfill area. The carbon emissions calculation involves the following steps:

#### Component lifecycle carbon emissions

Calculate carbon emissions associated with the lifecycle of each component, including production, transportation, installation, and disposal. This includes photovoltaic modules, inverters, supporting structures, and other components.

#### Transportation emissions

Consider emissions generated during the transportation of components from production locations to the construction site, taking into account factors like the type of freight vehicles, distance, and fuel efficiency.

#### Energy use carbon emissions

Estimate carbon emissions resulting from the system's energy requirements during the operational phase, considering factors such as the source of electricity and energy conversion efficiency.

#### Maintenance carbon emissions

Record and evaluate carbon emissions resulting from maintenance activities, such as fuel consumption by maintenance vehicles.

These data will be synthesized to calculate the full lifecycle carbon emissions of the system, considering emissions throughout the construction, operation, and maintenance phases.

### Analysis of carbon emission reduction benefits: environmental and economic aspects

Performing an analysis of carbon emission reduction benefits is crucial for evaluating the environmental and economic advantages of the photovoltaic system in the Macau construction waste landfill area. This analysis comprises the following key elements:

#### Environmental benefits

Quantify the reduction in carbon emissions achieved by the system in comparison to traditional energy sources. This assessment facilitates a deeper understanding of the system's positive impact on climate change and air quality.

#### Economic benefits

Evaluate the economic benefits linked to the photovoltaic system, including energy savings, maintenance costs, and potential income from electricity sales. This assessment assists in determining the system's sustainability and economic feasibility.

#### Return on investment (ROI)

Determine the return on investment to assess the economic viability of the project. This evaluation includes integrating both the initial investment and the system's operational lifespan.

By conducting these analyses and assessments, we will gain a comprehensive understanding of the potential impact of the photovoltaic system in the Macau construction waste landfill area, particularly regarding carbon emissions reduction and economic benefits. This supports sustainable decision-making and policy development.

## Conclusion

### Summary of key research findings

In this study, we conducted a full lifecycle carbon emissions calculation and a carbon emission reduction benefit analysis for the photovoltaic system within the Macau construction waste landfill area. The following summarizes the key research findings:

#### Carbon emission calculation

Utilizing comprehensive data and models, we calculated the full lifecycle carbon emissions of the photovoltaic system in the Macau construction waste landfill area. This calculation includes emissions throughout the component lifecycle, transportation emissions, energy use emissions, and maintenance emissions^31^.

If calculated based on the carbon emissions model in this study, the CO^2^ payback period for the system is approximately 2.5 years. Assuming only an industrial electricity price of 0.81 MOP/(kW/h) is considered, the project can recover the entire project cost by the 13th year. Furthermore, if carbon emission trading amounts are taken into account, using the example of the average daily transaction price on the Shanghai Carbon Exchange, the project can recoup the total investment by the 8th year. This effectively overturns the misconception of the photovoltaic industry being "high-energy-consuming" and "high-polluting."

#### Carbon emission reduction benefits

We found that the photovoltaic system, relative to traditional energy sources, can significantly reduce carbon emissions. This has a positive impact on enhancing environmental quality, addressing climate change, and reducing carbon footprints.

#### Economic benefits

The economic benefit analysis suggests that the photovoltaic system within the Macau construction waste landfill area not only contributes to environmental protection but also offers substantial economic returns through energy savings and potential electricity sales.

### Answers and insights into research questions

The research questions involved the implementation of a photovoltaic system within a construction waste landfill area and the potential impacts of the system in terms of carbon emissions reduction and economic benefits. Our study provided answers to these questions and offered the following insights:

#### Sustainability

The construction of the photovoltaic system has a positive impact on the environment, contributing to carbon emissions reduction, improved environmental quality, and the promotion of sustainable development.

#### Economic viability

The photovoltaic system not only provides environmental benefits but also presents potential economic returns. This can incentivize investors to engage in photovoltaic projects within construction waste landfill areas.

#### Policy support

The research results emphasize the significance of policy support and the encouragement of renewable energy projects. Governments and relevant stakeholders can implement measures such as providing tax incentives, introducing carbon pricing policies, and supporting renewable energy certificate programs to drive the development of photovoltaic systems.

#### Replicability

The methods and results of this study can serve as valuable guidance for other regions or areas with similar environmental conditions, particularly waste landfill areas, regarding the replicability of photovoltaic systems in managing construction waste and reducing carbon emissions.

### Application of research findings and policy recommendations

Based on our research, we propose the following applications and policy recommendations:

#### Promotion of photovoltaic projects

Government authorities and relevant stakeholders should proactively promote projects involving the installation of photovoltaic systems within construction waste landfill areas. This can be achieved by offering financial incentives, streamlining approval processes, and providing technical support.

#### Implementation of carbon pricing policies

Governments can implement carbon pricing policies to encourage carbon emissions reduction and the development of renewable energy projects. This will provide greater economic incentives for photovoltaic systems.

#### Integration into energy policies

Include photovoltaic systems within comprehensive energy policies to ensure their integration with the grid and other renewable energy sources. This will contribute to enhanced system availability and sustainability.

#### Monitoring and reporting

Builders and operators should establish systematic monitoring and reporting mechanisms to track the system's performance, carbon emissions, and economic benefits. This will assist in system maintenance and provide reliable data to support policy decisions.

The research findings can be utilized to guide future decisions regarding the implementation of photovoltaic systems within landfill areas, promoting sustainable development and carbon emissions reduction.

## Data Availability

We regret that we are unable to disclose the original data due to privacy and ethical constraints. Access to the original data is governed by strict privacy and ethical guidelines. However, permission to access this original data can be granted upon request to the corresponding author, pending approval from the Ethics Committees of Macau Fung Chak Engineering Company Limited.
